# *MKX-AS1* Gene Expression Associated with Variation in Drug Response to Oxaliplatin and Clinical Outcomes in Colorectal Cancer Patients

**DOI:** 10.3390/ph16050757

**Published:** 2023-05-17

**Authors:** Ricardo D. Gonzalez, George W. Small, Adrian J. Green, Farida S. Akhtari, Alison A. Motsinger-Reif, Julia C. F. Quintanilha, Tammy M. Havener, David M. Reif, Howard L. McLeod, Tim Wiltshire

**Affiliations:** 1 Division of Pharmacotherapy and Experimental Therapeutics, UNC Eshelman School of Pharmacy, University of North Carolina at Chapel Hill, Chapel Hill, NC 27599, USA; rgonzalez1@unc.edu (R.D.G.);; 2 Center for Pharmacogenomics and Individualized Therapy, University of North Carolina at Chapel Hill, Chapel Hill, NC 27599, USA; 3 Department of Biological Sciences, North Carolina State University, Raleigh, NC 27606, USA; 4 Bioinformatics Research Center, North Carolina State University, Raleigh, NC 27606, USA; 5 Biostatistics and Computational Biology Branch, Division of Intramural Research, National Institute of Environmental Health Sciences, Research Triangle Park, NC 27709, USA; 6 Clinical Development, Foundation Medicine, Boston, MA 02115, USA; 7 Structural Genomics Consortium and Division of Chemical Biology and Medicinal Chemistry, Eshelman School of Pharmacy, University of North Carolina at Chapel Hill, Chapel Hill, NC 27599, USA; 8 Predictive Toxicology Branch, Division of Translational Toxicology, National Institute of Environmental Health Sciences, Research Triangle Park, NC 27709, USA; 9 Center for Precision Medicine and Functional Genomics, Utah Tech University, St. George, UT 84770, USA

**Keywords:** precision medicine, colorectal cancer, pharmacogenomics, oxaliplatin, lncRNA’s, antisense

## Abstract

Oxaliplatin (OXAL) is a commonly used chemotherapy for treating colorectal cancer (CRC). A recent genome wide association study (GWAS) showed that a genetic variant (rs11006706) in the lncRNA gene *MKX-AS1* and partnered sense gene *MKX* could impact the response of genetically varied cell lines to OXAL treatment. This study found that the expression levels of *MKX-AS1* and *MKX* in lymphocytes (LCLs) and CRC cell lines differed between the rs11006706 genotypes, indicating that this gene pair could play a role in OXAL response. Further analysis of patient survival data from the Cancer Genome Atlas (TCGA) and other sources showed that patients with high *MKX-AS1* expression status had significantly worse overall survival (HR = 3.2; 95%CI = (1.17–9); *p* = 0.024) compared to cases with low *MKX-AS1* expression status. Alternatively, high *MKX* expression status had significantly better overall survival (HR = 0.22; 95%CI = (0.07–0.7); *p* = 0.01) compared to cases with low *MKX* expression status. These results suggest an association between *MKX-AS1* and *MKX* expression status that could be useful as a prognostic marker of response to OXAL and potential patient outcomes in CRC.

## 1. Introduction

Colorectal cancer (CRC) is a leading cause of cancer deaths among both men and women in the US. Incidence rates can differ by race and ethnicity, with higher rates among African Americans and Ashkenazi Jews and lower rates among Asian Americans [1]. A significant number of CRC patients will experience metastatic disease during treatment [2,3]. Despite progress in early detection and screening, CRC is still a significant health issue. The 5-year survival rate for CRC has improved from approximately 50% in the 1970s to 64% by 2015, but it drops to just 14% when it spreads to other organs [1,4].

Oxaliplatin (OXAL) is still widely used in combination regimens as a chemotherapeutic agent in CRC due to its effectiveness and tolerability, even with the advent of treatments such as monoclonal antibodies [5]. CRC treatments include 5-fluorouracil (5-FU), irinotecan, the FOLFOX regimen (folinic acid, fluorouracil, and OXAL), and biologic agents, such as bevacizumab, cetuximab, and panitumumab, used for metastatic CRC and adjuvant therapy [2,5,6,7,8,9,10,11]. Despite being effective, resistance remains a major issue. Factors that contribute to the development of CRC, treatment resistance, and poor survival rates include age, family history, inflammatory bowel disease, lifestyle factors, and certain inherited genetic factors and conditions [2,3,5,8,12].

High throughput drug screens utilizing cell line models assessed the sensitivity and resistance of different compounds and identified both germline and somatic variants that influence drug response to improve our understanding of cancer biology and novel therapeutic strategies [13,14,15,16]. The 1000 Genomes Project’s collection of immortalized B lymphocytes (LCLs) and the use of cancer cell lines provide resources for studying the impact of human genetics on drug response and disease, allowing for large-scale gene expression and genome-wide association studies across diverse, multinational populations. These cell line models offer valuable in vitro models to gain insights into cancer biology and treatment responses [16,17,18,19,20].

Pharmacogenetics in CRC previously identified various genetic factors that influence the drug response of various cancer treatments [5,21,22,23,24,25,26,27]. In this study, we explored the potential impact of a genetic factor on the response to OXAL treatment from a high-throughput assay using LCLs [13,28]. Based on prior findings indicating a significant correlation between a *MKX-AS1* (Mohawk Homeobox Antisense RNA 1) variant and response to OXAL, our goal was to further investigate the involvement of *MKX-AS1* in the response of OXAL in CRC.

## 2. Results

### 2.1. GWAS Analysis

**Figure 1:**
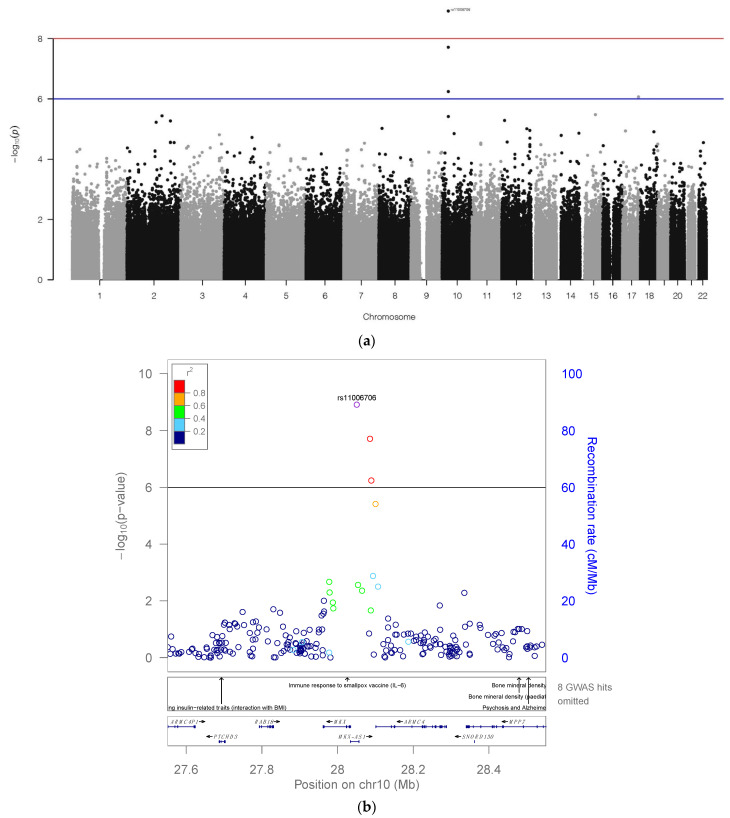
(**a**) Manhattan plot −log10 *p*-values for the GWAS for OXAL. Solid blue line indicates threshold for suggestive significance and redline indicates genome wide significance. (**b**) Peak on chromosome 10 has one SNP surpassing the genome-wide significance. Locus Zoom plot showing region surrounding rs11006706. Colors are matched to lead SNP with which it is in highest linkage disequilibrium (LD).

The results of a high-throughput dose-response screen of 44 anticancer drugs conducted on the 1000 Genome collection of LCLs by Akhtari et al. showed one significant SNP association with OXAL response, as indicated on the Manhattan plot (Figure 1a) [13]. This association was centered on chromosome 10, and the locus zoom plot of the region’s genes (Figure 1b) showed that the SNP rs11006706 (*p*-value = 1.22 × 10^−9^) exceeded genome-wide significance (*p* < 10 × 10^−8^). rs11006706 is an intronic SNP located in the long non-protein-coding antisense RNA (lncRNA) *MKX-AS1*. Although rs11006706 was predicted to have a neutral phenotype, a secondary linkage disequilibrium (LD) analysis revealed a SNP—rs11006701—which was not detected in the GWAS but is in complete LD (R2 = 1.0) with rs11006706 and predicted to be deleterious (Supplement Table S1).

Given the limited information on *MKX-AS1*, studying any lncRNA remains difficult due to their diverse and complex functions. However, there is evidence that naturally occurring antisense transcripts can affect the expression and processing of sense transcripts [29,30,31,32,33]. Thus, it is also crucial to examine the relationship between *MKX-AS1* and its partner, which is the sense *MKX* (Mohawk Homeobox) protein-coding gene, in relation to OXAL response. To assess the functional significance of rs11006706, we investigated the relationship between both *MKX-AS1* and *MKX* gene expression levels, and explored how the genotype is related to the response to OXAL dose.

### 2.2. MKX-AS1 and MKX Gene-Expression

**Figure 2:**
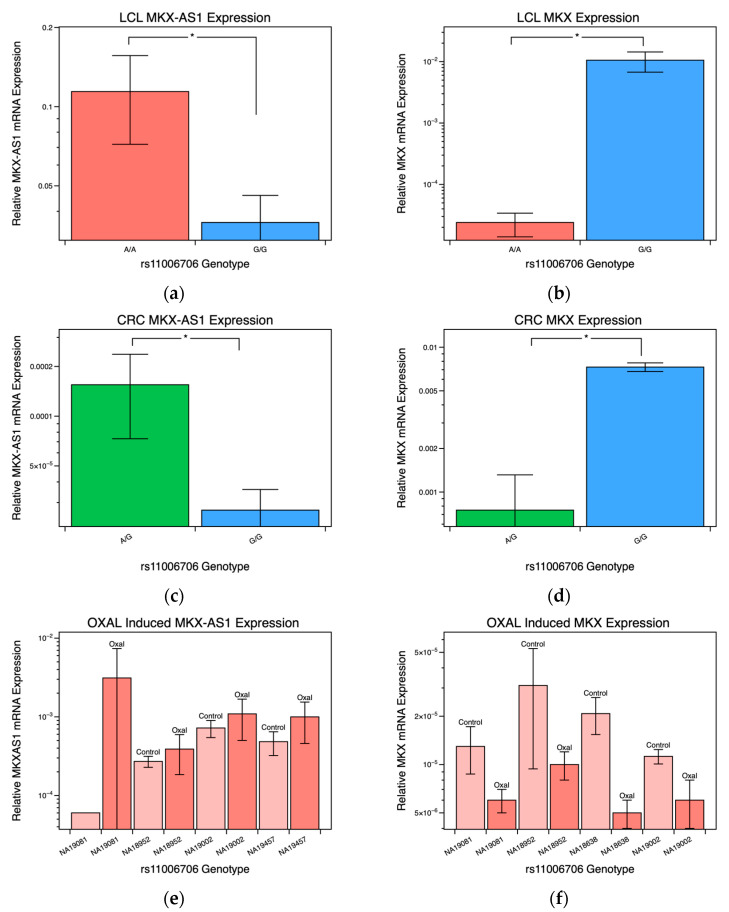

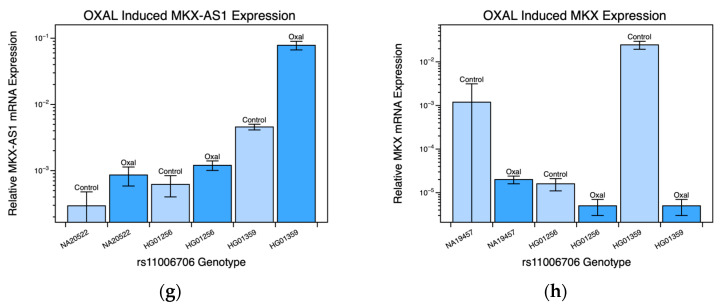
Associations transcriptional activity of *MKX-AS1* and *MKX* gene expression stratified using genotype rs11006706. (**a**) Relative *MKX-AS1* mRNA levels ± SE by genotype performed using qPCR in LCLs. (**b**) Relative *MKX* mRNA levels ± SE by genotype performed using qPCR in LCLs. (**c**) Relative *MKX-AS1* mRNA levels ± SE by genotype performed using qPCR in CRC cell lines. (**d**) Relative *MKX* mRNA levels ± SE by genotype performed using qPCR in CRC cell lines. Transcriptional activity of *MKX-AS1* and *MKX* in LCL cell lines measured following 72 h treatment with OXAL (darker shade) at the median concentration 5 mM used in cell viability screening. (**e**) AA genotyped LCLs *MKX-AS1* OXAL-induced expression using qPCR. (**f**) AA genotyped LCLs *MKX* OXAL-induced expression using qPCR. (**g**) GG genotyped LCLs *MKX-AS1* OXAL-induced expression using qPCR. (**h**) GG genotyped LCLs *MKX* OXAL-induced expression using qPCR. * *p*-value < 0.05.

We investigated the association of *MKX-AS1* and *MKX* gene expression levels stratified using rs11006706 genotype. We also examined the associations between baseline- and OXAL-induced expression levels using RT-qPCR after 72 hrs. In the LCLs, there were significant differences in baseline expression of the *MKX-AS1*, where the GG genotyped cells had lower expression compared to the AA genotyped cells (Figure 2a, *p* < 0.01). Conversely, for baseline expression of *MKX*, GG genotyped cells had higher expression compared to the AA genotyped cells (Figure 2b, *p* < 0.01). When investigating drug-induced expression between AA and GG genotypes, both groups expressed more *MKX-AS1* when treated with OXAL (Figure 2e,g). On the other hand, both groups expressed less *MKX* when treated with OXAL (Figure 2f,h).

To study the relationship between *MKX-AS1* and *MKX* on the effectiveness of OXAL in cancer cell lines, we instead used CRC cell lines. No CRC cells genotyped (n = 7) had an AA genotype present at rs11006706. Our results showed that CRC cells with a GG genotype had reduced *MKX-AS1* expression and elevated *MKX* expression, similar to what was observed in genotyped LCLs (Figure 2c,d). Other studies revealed that the expression of *MKX-AS1* and *MKX* were reduced in colorectal carcinoma compared to normal tissue. This trend was also evident in the comparison of LCL and CRC cell line expression levels. [34,35].

### 2.3. OXAL Dose Response

**Figure 3:**
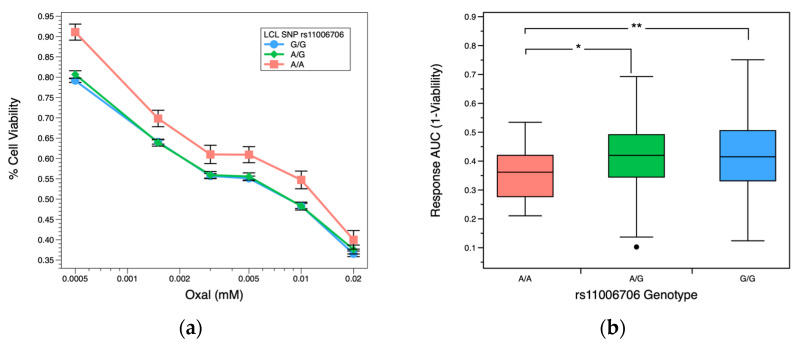

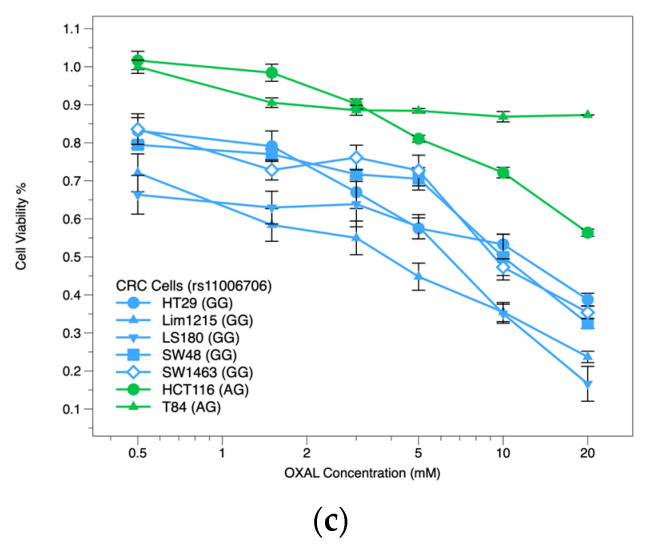
Associations for OXAL response data stratified by genotype rs11006706. (**a**) LCLs dose-response profiles for OXAL. Multiple cell lines represent each genotype, with a total of n = 644 cell lines; GG—453, GA—166, AA—25. Concentrations are on log scale. Viability mean ± SE. (**b**) Box plot of AUC response area for OXAL by genotype. A higher AUC indicates higher drug response to OXAL treatment. (**c**) CRCs dose-response profiles for OXAL. Concentrations are on log scale. Cell viability mean ± SE. * *p*-value < 0.05, ** *p*-value < 0.005.

To further evaluate the functional impact of rs11006706, we studied the connection between rs11006706 genotype of *MKX-AS1* and how it relates to the response to OXAL dose. Figure 3a shows LCLs dose response profiles stratified by genotype. Individuals with AA genotype have higher cell viability when exposed to all concentrations of OXAL tested compared to AG and GG genotypes. Similarly, Figure 3b shows that the AA genotype cells have lower area under the curve (AUC) responses, implying a reduction in drug response and resistance to OXAL than in the GG genotype cell lines (*p* = 0.013). The only genotypes present in the CRC cells treated with OXAL were 2-AG and 5-GG (Figure 3c), consistent with the results observed in the LCLs. The AG genotyped CRC cells showed increased cell viability when exposed to all levels of OXAL tested compared to the GG genotyped cells.

### 2.4. MKX siRNA Knockdown

**Figure 4:**
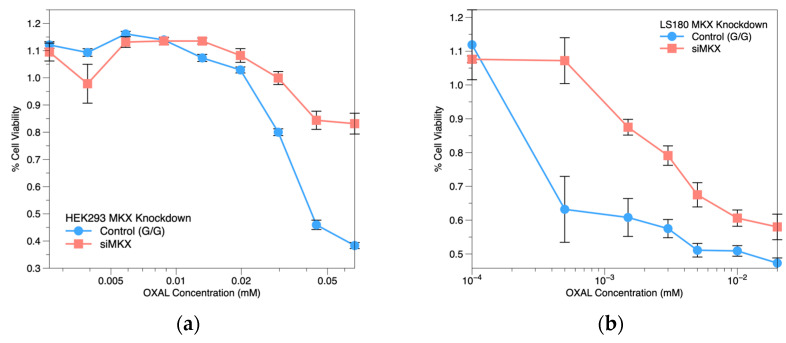
*MKX* siRNA knockdown OXAL dose−response profile: (**a**) HEK293 cell line; (**b**) LS180 CRC cell line. Concentrations are on log scale. Cell viability ± SE.

Our results showed that as *MKX-AS1* expression increased, *MKX* expression decreased in response to OXAL. To further examine the role of *MKX* in OXAL sensitivity in CRC cells, we conducted knockdown experiments using siRNAs to reduce *MKX* expression in HEK293 (GG) and LS180(GG) cells. We found that *MKX* knockdown resulted in decreased gene and protein expression, as confirmed via qPCR and western blotting. The HEK293 *MKX* knockdown cells showed a more resistant phenotype to OXAL, as evidenced via a significant increase in cell viability and a higher IC50 (1.32 ± 0.03 mM) compared to the control cells (0.03 ± 0.01 mM) (Figure 4a). The LS180 *MKX* knockdown cells also showed a more resistant phenotype to OXAL, as evidenced via a significant increase in cell viability and a higher IC50 (0.04 ± 0.0042 mM) compared to the control cells (0.01 ± 0.0006 mM) (Figure 4b).

### 2.5. MKX-AS1 and MKX Expression Is Associated with Poor Clinical Prognosis

**Figure 5:**
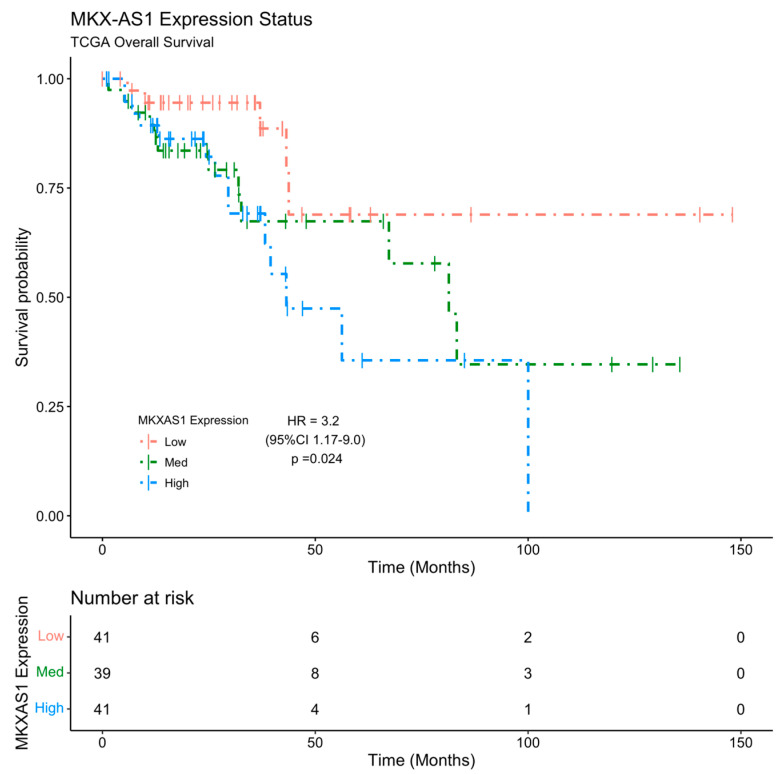
Kaplan–Meier curve of OS for TCGA CRC patients’ cohorts based on *MKX-AS1* gene expression status. Cox Proportional Hazards Model Hazard Ratio with 95% Confidence Interval and *p*-value is shown. Mean survival time for *MKX-AS1* expression status: high = 56.8 ± 8.31 months; medium = 83.1 ± 13.16 months; low = 113.2 ± 14.44 months.

To investigate whether *MKX-AS1* and *MKX* expression status is associated with patient outcomes, a Kaplan–Meier survival analysis was assessed for overall survival (OS) of patients with CRC tumors and gene expression from the Cancer Genome Atlas (TCGA) dataset. We found that *MKX-AS1* expression showed significant association with survival outcomes. TCGA cases with high *MKX-AS1* expression status had significantly worse overall survival (HR = 3.2; 95%CI = (1.17–9); *p* = 0.024) compared to cases with low *MKX-AS1* expression status (Figure 5).

**Figure 6:**
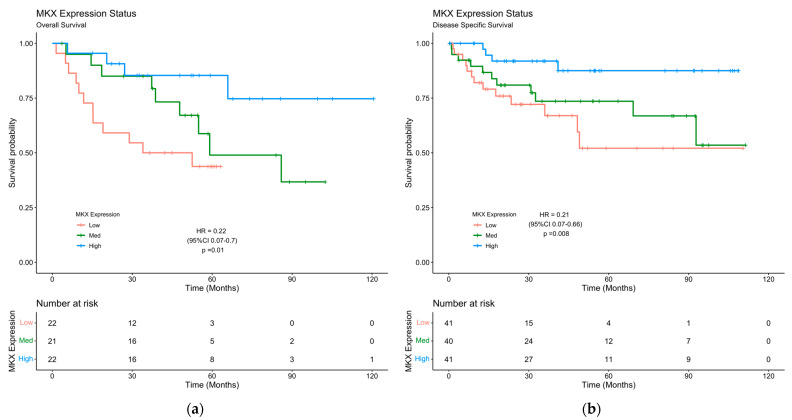
Kaplan–Meier curve of OS and DSS for CRC patients’ cohorts based on *MKX* gene expression status. Cox Proportional Hazards Model Hazard Ratio with 95% Confidence Interval and *p*-value is shown. (**a**) Mean survival time: high = 99.8 ± 9.27 months; medium = 74.5 ± 10.51 months; low = 63.1 ± 11.27 months. (**b**) Mean survival time: high = 100.4 ± 5.17 months; medium = 80.7 ± 7.57 months; low= 70.0 ± 9.09 months.

We also performed a Kaplan–Meier survival analysis to assess the association of *MKX* expression status on OS and disease-specific survival (DSS) outcomes from four public expression profiles of independent CRC datasets. GSE29623 and GSE38832 contained human gene expression profile data regarding solid tissues of CRC and prognostic survival information for analysis. The subgroup of CRC patients with high *MKX* expression status had significantly better OS (HR = 0.22; 95%CI = (0.07–0.7); *p* = 0.01) and DSS (HR = 0.21; 95%CI = (0.07–0.66); *p* = 0.008) survival (Figure 6).

To gain a deeper understanding of the correlation between OXAL and *MKX* regulation, we performed Kaplan–Meier survival analysis on the subset of patients who received OXAL as part of their treatment regimens, using the available data on OXAL treatment from two studies: GSE87211 and GSE72970. Our analysis revealed that there was no significant difference in survival outcomes between *MKX* expression subgroups in patients who received OXAL as part of their treatment regimen. We also examined MSI (Microsatellite Instability), which can be crucial in determining prognosis and guiding treatment decisions. However, in the CRC data available with MSI status, the association was not significant.

To add further clinical evidence, oxaliplatin treatment was also explored in clinical trials or off-label settings for other malignancies, including esophageal cancer. In addition, a regression analysis of TCGA Esophageal Adenocarcinoma patients revealed that patients with medium *MKX* expression status had significantly better overall survival (HR= 0.46; 95%CI = (0.218–0.99); *p* = 0.047) compared to cases with low *MKX* expression status. High *MKX* expression status also trended toward improved outcomes (Supplemental Figure S1).

## 3. Discussion

Personalized medicine places a major emphasis on using genetic information to design and customize treatments for individuals. In this study, we validate the relationship between *MKX-AS1* and *MKX* genes and the response to OXAL using LCLs and CRC cells. Following the discovery of a GWAS signal in the *MKX-AS1*-OXAL drug gene pair, we analyzed the *MKX-AS1* lncRNA gene and the *MKX* gene to explore their potential as markers of OXAL response and determine their functional roles.

The importance of lncRNAs in the regulation of gene expression, cellular processes, and disease development was established in recent studies [36,37,38,39]. *MKX-AS1* was only identified as a differentially expressed RNA in thyroid carcinoma, with no further information available [37,39]. Further exploration of the potential impact of *MKX-AS1* on *MKX* gene expression provided new insights into the role of *MKX* in cancer development [40,41,42,43,44]. One study showed a relationship between *MKX* methylation levels and the progression-free survival of ovarian cancer patients undergoing platinum chemotherapy [45].

The SNP rs11006706 within the *MKX-AS1* locus was identified as a genetic factor related to OXAL response based on the results of the GWAS. The association of rs11006706 with *MKX* gene expression and OXAL response was established through the study of both LCLs and CRC cell lines. The baseline *MKX-AS1* and *MKX* expression data revealed a significant difference between genotype groups. Cell lines of individuals with the AA variant showed significantly higher *MKX-AS1* expression and lower *MKX* expression compared to those with the GG genotype. The AA variant cell lines demonstrated a lower AUC response, implying a reduction and a less active drug response to OXAL, while the higher AUC response in the GG genotype cell lines indicated a more sensitive phenotype and a greater active drug response to OXAL, as evidenced by the dose-response curves. We observe that higher levels of *MKX-AS1* expression are correlated with increased cell viability when treated with OXAL in cell line models, implying that some of the variation between individuals may be influenced via *MKX-AS1* expression. Our results also reveal that higher *MKX-AS1* expression is connected to lower levels of *MKX* expression and vice versa. While using cell line model systems can be useful for testing drug response, we acknowledge that their limitations in representing the complex cellular microenvironment of human tumors make it challenging to determine whether the response is an accurate representation of efficacy or toxicity. Incorporating additional in vivo model systems, such as spheroids and animal models, is necessary to overcome these limitations. Spheroids can more accurately mimic the tumor microenvironment, providing a better assessment of drug response [46,47], while animal models can offer pre-clinical data on drug efficacy and toxicity in vivo [48,49]. Through incorporating these models in future studies, more comprehensive data on drug response can be obtained to inform clinical studies.

rs11006706 was not previously known to be a genetic risk factor. Further analysis of rs11006706 revealed a strong linkage with SNP rs11006701, which is located within a splicing region of the *MKX* gene. This discovery may help to explain the relationship between the rs11006706 genotype variants, *MKX-AS1* and *MKX* expression, and response to OXAL in CRC patients. These SNPs could indicate a potential haplotype effect. Additionally, the varying incidence rates and response variability seen in CRC patients may be partially described due to differences in rs11006706 minor allele frequencies (Supplemental Table S2) across ethnic groups, as African Americans tend to have a higher incidence of CRC, while Asian Americans tend to have a lower incidence of CRC [50,51].

As of now, the exact mechanism linking the SNP rs11006701 AA genotype to reduced oxaliplatin exposure remains unclear. However, we have identified several potential hypotheses based on our understanding of the *MKX-AS1* and *MKX* gene functions and their relationship with the rs11006706 genetic variant. The biological protein coding gene *MKX* is primarily involved in the development of tendons and ligaments and was not shown to be directly involved in drug metabolism or clearance. However, recent studies indicated that ncRNAs, such as *MKX-AS1*, may play important roles in the modulation of CRC proliferation and apoptosis [52,53,54]. In particular, specific ncRNAs were found to play crucial roles in the biology of colorectal cancer (CRC) via modulating cell proliferation and apoptosis through various signaling pathways, such as the Wnt/β-catenin and PI3K/Akt pathways [55,56]. Past studies showed *MKX* can interact with β-catenin and inhibit Wnt/β-catenin signaling, suggesting that *MKX* may act as a negative regulator of this pathway [57]. Consequently, a decrease in *MKX* expression, such as in individuals with the AA variant, can result in the sustained activation of Wnt/β-catenin signaling. This sustained activation can lead to the attenuated apoptotic behavior and sustained proliferative ability of colorectal cancer (CRC) cells and can also contribute to the reduced efficacy of oxaliplatin and an increased resistant phenotype in these cells. [58]. Thus, it appears that rs11006706 variants hold greater significance in CRC diagnosis and prognosis, and additional experimentation is necessary to establish the predictive value of this biomarker for oxaliplatin response. For a direct mechanism, it is possible that *MKX* could impact extra cellular matrix (ECM) genes in CRC cells, potentially affecting the response to chemotherapies, such as oxaliplatin [59,60,61,62,63]. Past studies suggested a potential role for *MKX* in extracellular matrix (ECM) remodeling and homeostasis; however, there is a lack of information regarding the specific effects of increased expression of the *MKX* gene on the ECM in CRC [64]. The structure and composition of the ECM can impact drug penetration and retention in the tumor microenvironment [65]. Thus, changes in ECM gene expression may influence the response to chemotherapy, while understanding the impact of *MKX* on the ECM could provide valuable insights into potential therapeutic strategies for CRC.

To translate the results of our study into real-life scenarios, we categorized CRC patients into three groups based on the expression levels of *MKX-AS1* expression status using gene expression data from TCGA database and *MKX* expression data from four separate datasets. High *MKX-AS1* expression levels (low *MKX* expression) were found to be a strong predictor of poor OS in TCGA patients, regardless of treatment. On the other hand, high *MKX* expression levels (low *MKX-AS1* expression) were associated with improved OS and disease-specific survival (DSS) in patients. Our findings of high *MKX* expression being associated with better survival outcomes were supported by similar observations available in the Human Protein Atlas (proteinatlas.org) in colon, liver, lung, and thyroid cancers. However, the results were opposite in urothelial, stomach, renal, and endometrial cancers, for which low *MKX* expression was associated with better survival outcomes [66,67,68].

Although OXAL-treated subgroups did not yield significant differences, it is important to note that our findings could potentially be confounded by various factors. Specifically, we observed that treatment with OXAL led to increased expression of *MKX-AS1* and decreased expression of *MKX*, which could mask the prognostic value of these genes in the survival analysis. Moreover, in CRC, OXAL is typically administered in combination with other anticancer agents, such as FOLFOXIRI, FOLFOX, or FOLFOX + BEVACIZUMAB regimens. The combination of different chemotherapies and mechanisms of action could further complicate the interpretation of survival results in OXAL-treated subgroups.

Oxaliplatin was previously investigated as a potential treatment option for several malignancies, including esophageal cancer, in clinical trials or off-label settings. Clinical evidence suggests that the expression of *MKX* and its association with oxaliplatin may have some efficacy in the treatment of esophageal cancer, but further research is needed to confirm these results. Additionally, mismatch repair (MMR) genes that help maintain genomic stability were found to play a significant role in the response to oxaliplatin. Therefore, assessing *MKX*/*MKX*AS1 expression, MMR status, and MSI can be crucial in determining prognosis and guiding treatment decisions for gastric, pancreatic, and esophageal cancers [69,70]. Patients with MSI-high (MSI-H) tumors tend to have better outcomes when treated with oxaliplatin-based chemotherapy regimens compared to those with microsatellite stable (MSS) tumors. The improved response in MSI-H tumors may be attributed to their increased sensitivity to DNA-damaging agents, such as oxaliplatin. This sensitivity is because MSI may lead to an accumulation of DNA damage and trigger cell death in cancer cells, providing a rationale for utilizing oxaliplatin in MSI-H tumors. In gastric cancer, deficient MMR (dMMR) and MSI are observed in approximately 15–30% of cases, whereas in pancreatic cancer, MMR gene mutations and MSI are less common, with a prevalence of 1–2% [69,70,71]. The prevalence of dMMR/MSI in esophageal cancer is also relatively low, ranging from 2–8%. Nonetheless, the presence of dMMR/MSI in these cancer types may have significant implications for prognosis and treatment options.

Our results suggest that both *MKX-AS1* and *MKX* expression levels may have greater prognostic relevance for CRC patients. However, the relationship between *MKX-AS1* and *MKX* expression, and their impact on OXAL response and survival outcomes in cancer patients, are not yet fully clear and require further investigation.

## 4. Materials and Methods

### 4.1. Cell Lines Culture and Genetic Data

We accessed 680 immortalized LCLs from the 1000 Genomes representing nine populations from different geographic and ethnic backgrounds. The genotype information, which involved SNP rs11006706, was obtained from the Illumina HumanOmni2.5 platform and processed following the methodology outlined by Abdo et al. [72]. The human CRC cell lines SW48, SW1463, DLD-1, T84, LS180, HCT116, HT-29, and LIM1215 were obtained from the American Type Culture Collection. The cells were kept frozen in liquid nitrogen, and after thawing, the CRC cell lines were cultured in their recommended complete medium. Each cell line was seeded on to 384-well plates with approximately 5000 cells per well and four replicates per plate. Cell count was measured using Trypan dye exclusion.

### 4.2. Dose Response

The OXAL viability data was available for all LCLs (n = 680) from a high-throughput anticancer agent screen developed by Akhtari et al. [13]. Background fluorescence control, 10% dimethyl sulfoxide (DMSO), and a drug vehicle control were included on every plate. Each cell line was subjected to all tested concentrations of each treatment for a period of 72 h. The fluorescence intensity was measured at EX535nm and EM595nm using AlamarBlue (Life Technologies, Carlsbad, CA, USA) and a Tecan F200 plate reader with iControl software (Version 1.6). The resulting raw fluorescence units (RFUs) were proportional to the concentration of living cells in each well and were used for measuring cell viability. Additional OXAL (NSC266046, Selleckchem) was dissolved in water or phosphate buffered saline (PBS) for drug-induced gene expression analysis.

### 4.3. SNP Genotyping and mRNA Expression

The DNA of cultured cells was extracted using the QIAamp DNA Mini Kit from Qiagen (Qiagen, Germantown, MD, USA) in accordance with the manufacturer’s instructions. The quantity and quality of the DNA were then evaluated using a nanodrop spectrophotometer (NanoDrop Technologies Inc., Wilmington, DE, USA). The genotyping for the rs11006706 (G > A) was performed with TaqMan™ allelic discrimination assay (Life Technologies, CA, USA) and, following the manufacturer’s instructions, using a CFX384 Real-Time PCR detection system (Bio-Rad Laboratories, CA, USA) with pre-designed TaqMan™ SNP Genotyping Assay, human (assay ID: C__32107647_10) (Life Technologies, CA, USA). Approximately 25 ng of gDNA was loaded per well on 384-well plates with TaqMan™ Genotyping Master Mix (Life Technologies, CA, USA) containing VIC and FAM reporter dyes. The samples were compared with the controls from the 1000 Genomes Project that had known SNPs. No-template reactions were also included as controls in each assay run.

Total RNA was extracted from cells using the RNeasy kit (Qiagen, MD, USA). The reverse transcription PCR was then performed using the Verso cDNA kit (Life Technologies, CA, USA), following the manufacturer’s instructions. The cDNA was amplified via real-time PCR using a CFX384 Real-Time PCR detection system (Bio-Rad Laboratories, Hercules, CA, USA) to determine *MKX* and *MKX-AS1* expression before and after OXAL treatment. A total reaction volume of 20 μL was used, which contained between 50–100 ng of cDNA. The expression was analyzed using pre-designed TaqMan^®^ Gene Expression Assay *MKX* (Hs00543190_m1) and *MKX-AS1* (Hs04406772_m1), as well as an endogenous control *GADPH* (Hs02786624_g1) (Life Technologies, CA, USA). The Ct-values for all samples were determined using FPK-PCR [73]. Using GAPDH as the endogenous housekeeping gene, the comparative CT method was employed to determine the levels of *MKX* and *MKX-AS1* mRNA. Triplicates for each cell sample were devised, and the mean (standard error) was calculated for each cell line.

### 4.4. shRNA Knockdown

HEK293 and T98G cells were transfected with Silencer^®^ Select siRNAs Predesigned small interfering RNA (siRNA) targeting human *MKX* (siRNA ID#: s49084) and Silencer Select Negative Control #1 and GAPDH control (Life Technologies, CA, USA) and Lipofectamine RNAiMAX (Life Technologies, CA, USA), according to the manufacture’s instruction. Following a 48-hour incubation time, cells were harvested post-transfection for analysis of protein and RNA levels. *MKX* knockdown was confirmed via western blot analysis. Cells were collected and washed with cold PBS before being lysed in RIPA lysis and extraction buffer reagents with added ™ Protease Inhibitor Cocktail kit (Life Technologies, CA, USA) on ice. The protein concentration was measured using BCA protein assay (Life Technologies, CA, USA). A total of 25 µg of protein was separated using SDS-PAGE electrophoresis and transferred onto a nitrocellulose membrane (Life Technologies, CA, USA). The membrane was then blocked and probed with a mouse anti-*MKX* antibody (MA526996, Life Technologies, CA, USA), and the loading control was performed using anti-GAPDH antibody (Life Technologies, CA, USA). The membrane was visualized using Alexa Fluor secondary antibody (Goat anti-mouse Alexa Fluor™ Plus 800 A32730) and a LiCOR Odyssey imaging system (LI-COR Biosciences, NE, USA). The protein density was analyzed using ImageJ and Fiji software (Version 2.9.0) [74,75].

### 4.5. Clinical Datasets

Our study used Kaplan–Meier curves for survival analysis, using data from various sources. We gathered the *MKX-AS1* and *MKX* gene expression data and corresponding clinical information from TCGA patients from the UCSC Xena database [76,77]. The datasets GSE29623 (65 samples), GSE38832 (122 samples), GSE72970 (124 samples), and GSE87211 (363 samples) were obtained from the NCBI GEO database and provided survival status and response information for CRC patients, including data on *MKX* gene expression and individuals undergoing an oxaliplatin-based chemotherapy regimen (http://www.ncbi.nlm.nih.gov/geo/. Accessed on 31 January 2023) [78,79]. From the GSE72970 datasets, we selected 40 patients who received the FOLFOXIRI, FOLFOX, or FOLFOX + BEVACIZUMAB regimen. From the GSE87211 datasets, we selected 166 patients treated with 5-FU/oxaliplatin/cetuximab/RT or 5-FU/oxaliplatin/RT [80,81].

### 4.6. Statistical Analysis

All experiments were performed in triplicates unless otherwise indicated. All results are expressed as the mean ± standard error. All statistical analyses were performed using R version 4.0.3 and RStudio. ANOVA, Student’s *t*-test, and post hoc Tukey, as appropriate, were used to analyze the significance of differences in gene expression levels. The R package ‘roc’ and “PharmacoGx” were used to calculate the area under the curve (AUC) and significance [82]. DataGraph (Version 4.6) (Visual Data Tools, Inc., Chapel Hill, NC, USA. https://www.visualdatatools.com/. accessed on 15 February 2022) was used to produce figures. Kaplan–Meier and Cox analyses were performed using the packages ‘survival’ and ‘survminer’ on RStudio. All *p* values were two-sided, and differences were defined as statistically significant for *p*-values < 0.05.

## 5. Conclusions

Our results found a relationship between rs11006706 and the expression levels of *MKX-AS1* and *MKX*, which can impact the response to OXAL. These findings suggest that the expression of both *MKX-AS1* and *MKX* could be valuable indicators of prognosis for CRC patients. Further study is required to understand the influence of other genetic factors on the role of *MKX-AS1* and *MKX* in cancer development and drug response, as results vary among different types of cancer. In general, our study highlights the potential of *MKX-AS1* and *MKX* as prognostic markers for guiding cancer treatment.

## Data Availability

The data that support the findings of this study are available from the corresponding author upon reasonable request.

## Supplementary Materials

The following supporting information can be downloaded at: https://www.mdpi.com/article/10.3390/ph16050757/s1, Supplemental Table S1. Predict *MKX-AS1* SNP rs11006706 [83,84,85,86,87,88]; Supplemental Table S2. Minor Allele Frequency for SNP rs11006706. Supplemental Figure S1. Kaplan–Meier curve of OS for the TCGA Esophageal Adenocarcinoma patient cohort based on the *MKX* gene expression status.
