# Supplementary material for: MKX-AS1 Gene Expression Associated with Variation in Drug Response to Oxaliplatin and Clinical Outcomes in Colorectal Cancer Patients
Source: Pharmaceuticals (Basel). 2023 May 17;16(5):757. doi: 10.3390/ph16050757 (PMC10222429; doi:10.3390/ph16050757)
Supplement: Supplementary file 1 [file pharmaceuticals-16-00757-s001.zip › pharmaceuticals-2305950-Supplementary.pdf]

pharmaceuticals

Supplemental Table S1. Predict MKX-AS1 SNP rs11006706. [83-88]

Genomic Position	rsID	REF	ALT	Genome Region	PredictSNP			CADD		
					Estimated effect	Score given by tool	Estimated expected accuracy	Estimated effect	Score given by tool	Estimated expected accuracy
chr10:28050840	rs11006706	G	A	ncRNA intronic	neutral	-1	0.88	neutral	4.663	0.8
chr10:27745700	rs11006701	G	C	ncRNA intronic	neutral	0.2806798	0.73	deleterious	11.62	0.69
chr10:27745700	.	G	A	ncRNA intronic	deleterious	0.42256593	0.91	deleterious	13.06	0.79
chr10:27745700	.	G	T	ncRNA intronic	deleterious	0.37735204	0.91	deleterious	11.89	0.69

DANN			FATHMM			FunSeq			GWAVA		
Estimated effect	Score given by tool	Estimated expected accuracy	Estimated effect	Score given by tool	Estimated expected accuracy	Estimated effect	Score given by tool	Estimated expected accuracy	Estimated effect	Score given by tool	Estimated expected accuracy
neutral	0.6220574	0.71	neutral	0.13318	0.89	neutral	1.65E-20	0.8	neutral	0.16	0.79
deleterious	0.8451956	0.62	neutral	0.18712	0.77	deleterious	1.79118225	0.64	neutral	0.29	0.7
deleterious	0.9631567	1	neutral	0.18737	0.77	deleterious	1.79117016	0.64	neutral	0.29	0.7
deleterious	0.9304451	0.96	neutral	0.20506	0.75	deleterious	1.79078091	0.64	neutral	0.29	0.7

Supplemental Table S2. Minor Allele Frequency for SNP rs11006706.

Global	Study-wide	5008	G=0.8235	A=0.1765
African	Sub	1322	G=0.9486	A=0.0514
East Asian	Sub	1008	G=0.6290	A=0.3710
Europe	Sub	1006	G=0.8897	A=0.1103
South Asian	Sub	978	G=0.786	A=0.214
American	Sub	694	G=0.824	A=0.176

* 1000Genomes.BioProject ID: PRJEB6930

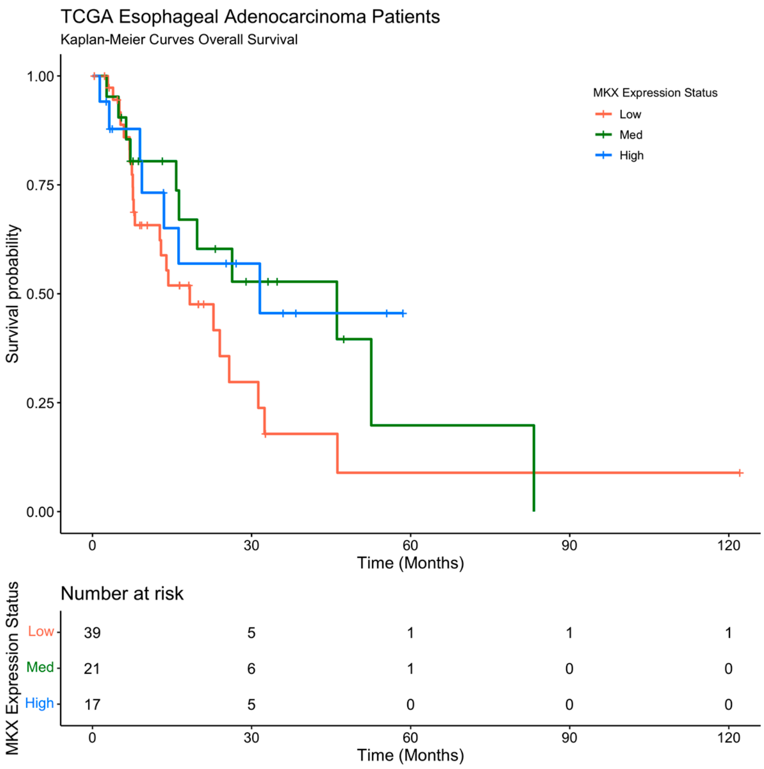
TCGA Esophageal Adenocarcinoma Patients
Kaplan-Meier Curves Overall Survival
Survival probability
1.00
0.75
0.50
0.25
0.00
0
30
60
90
120
Time (Months)
MKX Expression Status
Low
Med
High
Number at risk
MKX Expression Status
Low 39 5 1 1 1
Med 21 6 1 0 0
High 17 5 0 0 0

(a)

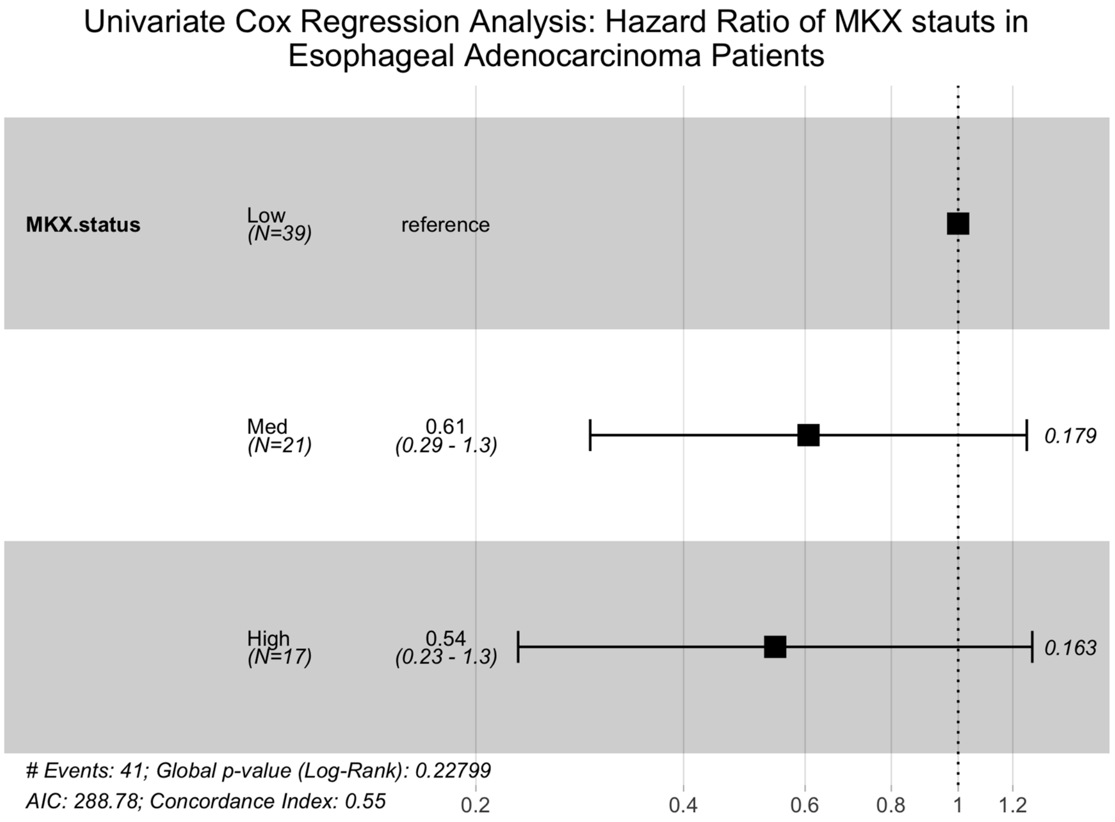
Univariate Cox Regression Analysis: Hazard Ratio of MKX stauts in Esophageal Adenocarcinoma Patients
MKX.status
Low (N=39) reference
Med (N=21) 0.61 (0.29 - 1.3) 0.179
High (N=17) 0.54 (0.23 - 1.3) 0.163
Events: 41; Global p-value (Log-Rank): 0.22799
AIC: 288.78; Concordance Index: 0.55
0.2
0.4
0.6
0.8
1
1.2

(b)

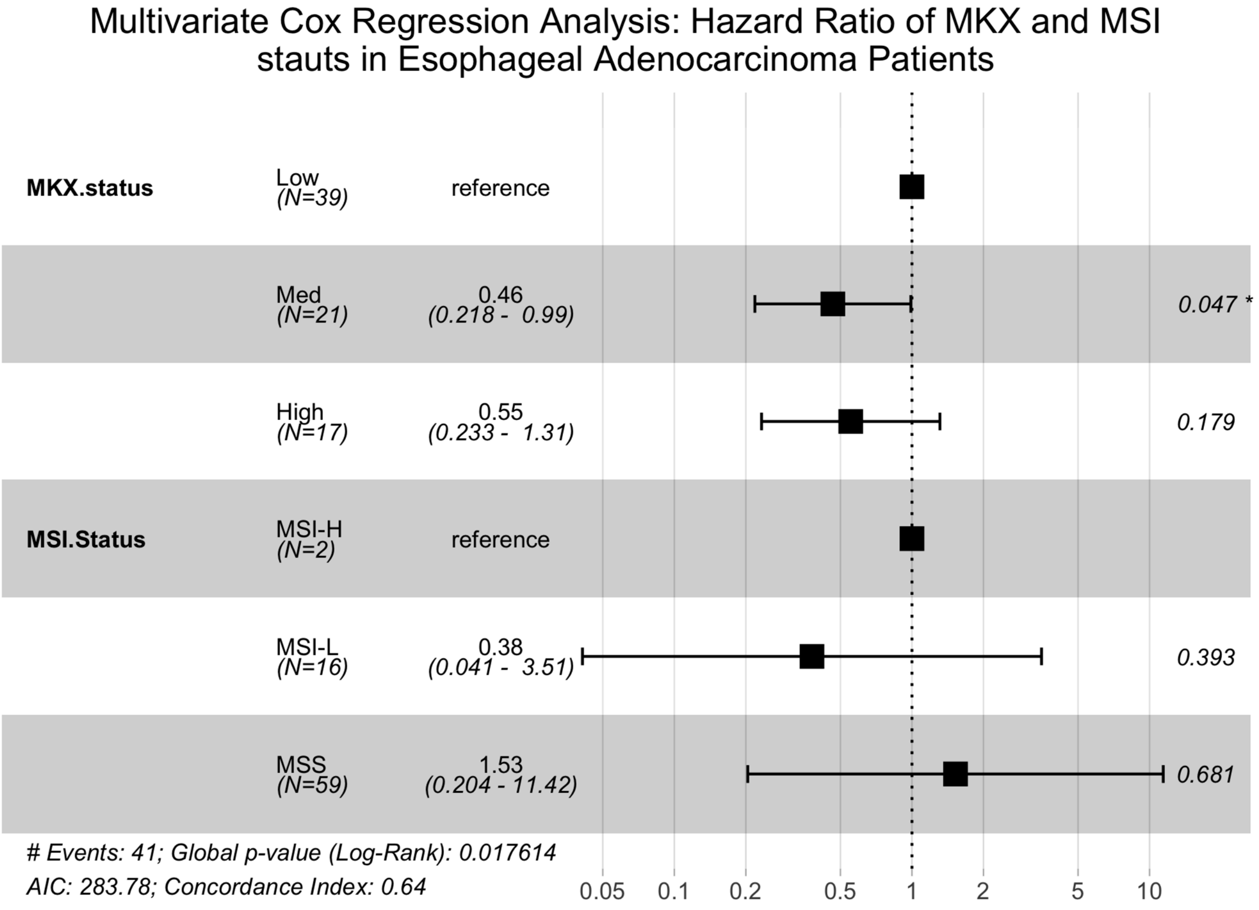

(c)

Figure S1. (a) Kaplan–Meier curve of OS for the TCGA Esophageal Adenocarcinoma patient cohort based on the *MKX* gene expression status. Cox Proportional-Hazards Model Hazard Ratio with 95% Confidence Interval and p-value is shown. **(b)** Forest plot showing univariate Cox regression analysis: hazard ratio of MKX status in TCGA Esophageal Adenocarcinoma patient cohort. **(c)** Forest plot showing the multivariate Cox regression analysis: hazard ratio of MKX and MSI (Microsatellite Instability) status in TCGA Esophageal Adenocarcinoma patient cohort. MSS- Microsatellite stable; MSI-L, Low levels of MSI; MSI-H, High levels of MSI.

References

83. Rentzsch, P.; Schubach, M.; Shendure, J.; Kircher, M. CADD-Splice-improving genome-wide variant effect prediction using deep learning-derived splice scores. *Genome Med.* **2021**, *13*, 31, doi:10.1186/s13073-021-00835-9.
84. Bendl, J.; Musil, M.; Štourač, J.; Zendulka, J.; Damborský, J.; Brezovský, J. Predictsnp2: A unified platform for accurately evaluating SNP effects by exploiting the different characteristics of variants in distinct genomic regions. *PLoS Comput. Biol.* **2016**, *12*, e1004962, doi:10.1371/journal.pcbi.1004962.
85. Bahcall, O. FunSeq for cancer genomics. *Nat. Genet.* **2013**, *45*, 1273–1273, doi:10.1038/ng.2819.
86. Ritchie, G.R.S.; Dunham, I.; Zeggini, E.; Flicek, P. Functional annotation of noncoding sequence variants. *Nat. Methods* **2014**, *11*, 294–296, doi:10.1038/nmeth.2832.
87. Quang, D.; Chen, Y.; Xie, X. DANN: a deep learning approach for annotating the pathogenicity of genetic variants. *Bioinformatics* **2015**, *31*, 761–763, doi:10.1093/bioinformatics/btu703.
88. Shihab, H.A.; Gough, J.; Mort, M.; Cooper, D.N.; Day, I.N.M.; Gaunt, T.R. Ranking non-synonymous single nucleotide polymorphisms based on disease concepts. *Hum Genomics* **2014**, *8*, 11, doi:10.1186/1479-7364-8-11.

Disclaimer/Publisher's Note: The statements, opinions and data contained in all publications are solely those of the individual author(s) and contributor(s) and not of MDPI and/or the editor(s). MDPI and/or the editor(s) disclaim responsibility for any injury to people or property resulting from any ideas, methods, instructions or products referred to in the content.
